# Computational modeling of oxygen dynamics in port-wine stain photodynamic therapy: treatment outcome optimization and pain management

**DOI:** 10.1117/1.JBO.31.2.028001

**Published:** 2026-02-03

**Authors:** Yijia Li, Qin Li, Xiaoming Hu

**Affiliations:** Beijing Institute of Technology, School of Medical Technology, Beijing, China

**Keywords:** photodynamic therapy, transcutaneous oxygen delivery, port-wine stains

## Abstract

**Significance:**

Port-wine stains (PWSs) are congenital capillary malformations with the incidence of in newborns of ∼0.8% to 2.1%. Hematoporphyrin monomethyl ether-mediated photodynamic therapy (HMME-PDT) has been widely applied in China for PWS. However, there remains substantial room for improvement in both the phototherapeutic selectivity coefficient (PSC) and pain management.

**Aim:**

We investigated the feasibility of modulating transcutaneous oxygen delivery during photodynamic therapy of PWS to enhance therapeutic efficacy and reduce pain.

**Approach:**

A three-dimensional (3D) computational biophysical model was employed to elucidate the mechanisms through which transcutaneous oxygen modulation enhances the therapeutic efficacy of HMME-PDT and improves pain management. The model was constructed to simulate the light propagation, photosensitizer kinetics, oxygen diffusion, and reactive oxygen species (ROS) generation. A treatment optimization strategy based on epidermal oxygen regulation was proposed and evaluated in computational studies. The spatiotemporal distributions of singlet oxygen under normoxic, hypoxic, and anoxic conditions were evaluated, and their effects on treatment-induced pain and lesion-targeted cytotoxicity were analyzed.

**Results:**

Computational analysis showed that compared with normoxic conditions, hypoxia and anoxia significantly enhanced PSC, with improvements of 48% and 61%, respectively. Furthermore, these oxygen-modulated regimens attenuated treatment-associated pain, reducing photochemical pain duration of 17% (hypoxia) and 30% (anoxia). Choosing the right combination of light source irradiance and surface oxygen supply rate amplified therapeutic performance and patient comfort, achieving a 213% increase in PSC and a 57% reduction in photochemical pain duration. These findings establish a mechanistic framework for advancing precision PDT protocols with minimized iatrogenic discomfort.

**Conclusions:**

Established in this computational study, strategic epidermal oxygen restriction critically augments PDT PSC while improving patient tolerance. Computational modeling demonstrates that controlled epidermal hypoxia spatially redistributes oxygen gradients, thereby suppressing superficial ROS generation in nontargeted epidermal layers and selectively concentrating ROS within PWS vasculature. This dual mechanism—simultaneously enhancing therapeutic precision and attenuating treatment-induced pain—presents a pioneering strategy centered on an active oxygen control strategy for enhancing HMME-PDT clinical outcomes. Future research will progress from preclinical validation in animal models to clinical studies to evaluate the therapeutic efficacy and translational potential of this strategy.

## 1 Introduction

Port-wine stains (PWS), also known as nevus flammeus or capillary malformations, are common congenital vascular anomalies. The incidence of PWS in newborns ranges from ∼0.8% to 2.1% across different countries.^1^ These lesions result from capillary dilation within the dermis and typically present at birth as flat pink or red patches on the skin. Over time, the lesions may darken to deep red or purple, and ∼70% of patients experience lesion thickening or nodular transformation by the age of 40.^2^ PWS can occur in isolation or as part of various vascular syndromes such as Sturge–Weber syndrome, often accompanied by soft tissue and skeletal hypertrophy, choroidal hemangioma, glaucoma, epilepsy, and other structural or functional abnormalities.^3^

Currently, the main treatment options for PWS include pulsed dye laser (PDL) and vascular photodynamic therapy (V-PDT). PDL is considered the gold standard for PWS treatment. Its principle relies on the theory of selective photothermolysis: Selective photothermolysis is achieved through irradiation at hemoglobin-specific wavelengths, where oxyhemoglobin absorption generates localized photothermal energy. This energy induces targeted photocoagulation of pathological vasculature via spatial confinement of thermal damage by optimizing laser pulse duration to align with the target-specific thermal relaxation time. Despite being the first-line therapy, only ∼10% to 20% of patients achieve complete blanching after PDL treatment,^4^^,^^5^ and ∼20% exhibit resistance to PDL.^6^

By contrast, V-PDT is a precision modality mediated through spatiotemporal control of three core elements: photosensitizer bio-distribution, wavelength-specific light irradiation, and dynamic oxygen gradients. Hemoporfin (HMME), a new generation porphyrin-based photosensitizer, offers better lesion uptake and shorter light-avoidance periods compared with first-generation agents and has gradually become the most widely used photosensitizer for PWS treatment.^7^ The typical hematoporphyrin monomethyl ether-mediated photodynamic therapy (HMME-PDT) protocol involves: a light power density of 80 to $100\;\mathrm{mW}/cm^{2}$,^8^ a drug dosage at 5  mg/kg,^9^ an administration for 5 to 20 min,^10^ and a treatment duration of 15 to 40 mi. In recent years, HMME-PDT has been widely adopted as an alternative for PDL-refractory PWS cases,^11^^–^^13^ though several challenges regarding its efficacy and safety remain.

First, PDT is often associated with intense pain during treatment, described as pricking, burning, or tearing sensations,^14^^,^^15^ which affects patient compliance and is a major concern in clinical practice.^10^^,^^16^ This pain primarily originates from free nerve endings in the papillary dermis, which are highly sensitive to nociceptive stimuli. Studies have shown that nociceptors are particularly responsive to rapid changes in reactive oxygen species (ROS), and elevated ROS levels are closely linked to neuronal damage and pain sensitization.^17^^,^^18^ Second, due to the selective distribution of oxygen and light, PDT often results in incomplete vascular damage and nonspecific epidermal injury. Clinically, increasing light doses or photosensitizer concentrations is commonly used to enhance selectivity, but this also increases the risk of pain and nonselective oxidative damage to adjacent epidermal/dermal structures.

Most existing studies focused on optimizing PDT parameters from an optical perspective. Jacques^19^ summarized the impact of tissue optical properties on light dose distribution in PDT. Yassine^20^ simulated the effects of various light source configurations and wavelengths on PDT for spinal metastases. Naiyan Huang et al.^21^ investigated the influence of laser power density on PDT efficacy for PWS. However, these strategies primarily address passive adjustment of light and photosensitizer dosage, failing to address the critical issue of ROS fluctuations induced by dynamic oxygen gradients during therapy.

In this study, a finite element model was constructed to systematically analyze the spatiotemporal distribution of light, oxygen, and ROS in tissue during PDT. By modulating oxygen levels at the skin surface, this study assessed how oxygen dynamics affect photochemical damage selectivity and pain. The simulation results showed that ROS concentrations remained within physiologically acceptable thresholds as reported in the literature,^22^^,^^23^ providing a pioneering strategy centered on active oxygen control for optimizing HMME-PDT clinical outcomes.

## 2 Materials and Methods

### 2.1 Geometric Model

**Fig. 1:**
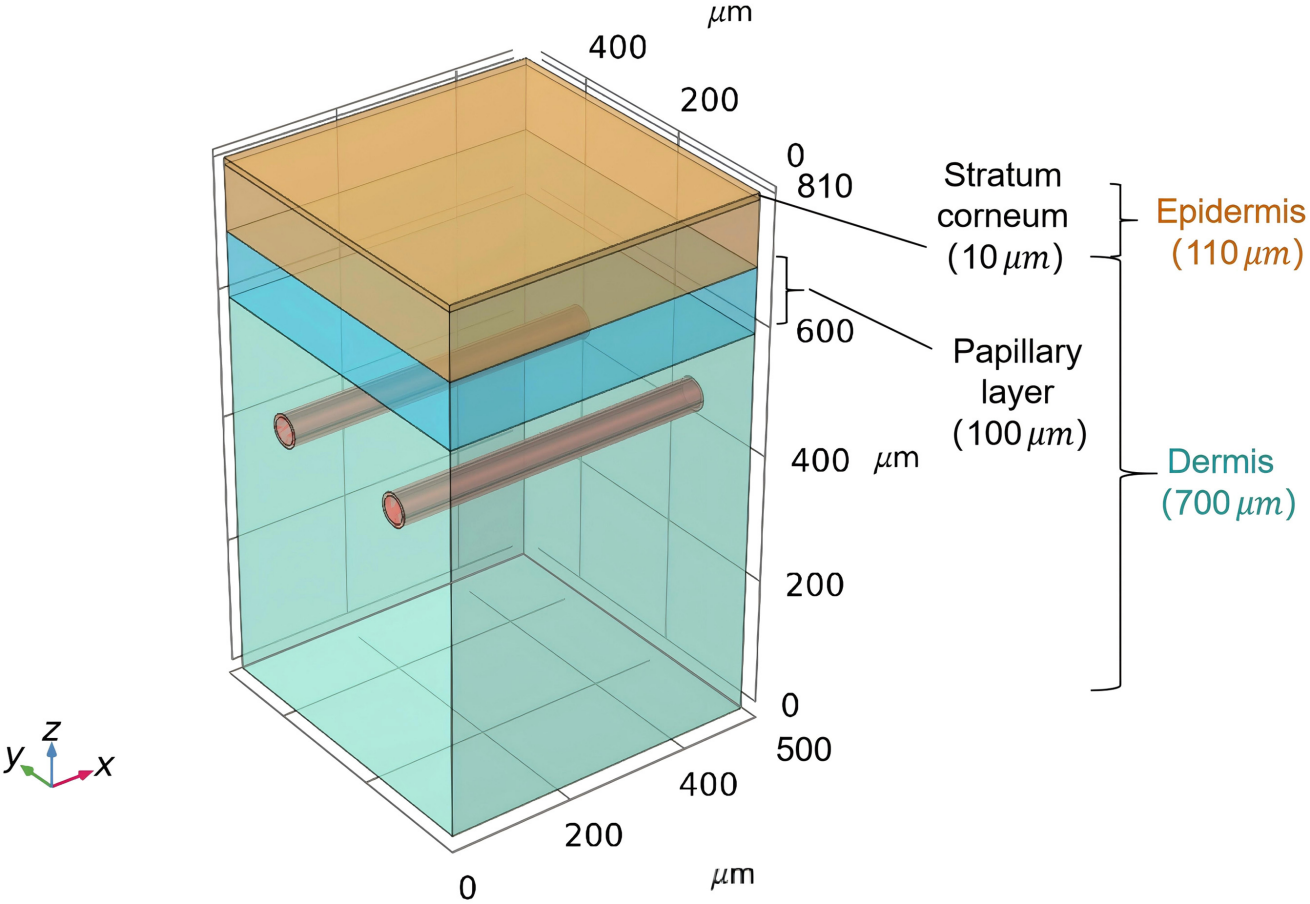
Geometric model for simulation.

In this study, a three-dimensional multiphysics model integrating light transport, oxygen dynamics, and hemodynamics is established to simulate PWS-PDT. The computational domain (Fig. 1) reconstructs an anatomically representative multilayered skin architecture: a 110  μm-thick epidermis (10  μm stratum corneum) overlying a 700  μm dermal compartment subdivided into papillary (100  μm) and reticular layers, consistent with histological benchmarks.^24^^–^^26^ Two parallel X-axis-aligned capillaries (central depth: 360  μm) with luminal diameter (40  μm), wall thickness (5  μm), and inter-capillary spacing (250  μm center-to-center) were embedded to mimic PWS vasculature. Pulsatile blood flow (500  μm/s peak velocity) was prescribed from arteriolar inlet to venular outlet, emulating physiological perfusion characteristics. The mathematical model comprised four sub-models: light transport, photosensitizer diffusion, oxygen diffusion, and singlet oxygen generation. Aligning with clinical practice, a treatment duration of 20 min (1200 s) is adopted to establish clinical relevance. Meanwhile, extended simulation durations are considered to analyze the trends in the temporal evolution of the photodynamic process.

### 2.2 Light Propagation in Biological Tissues

A typical 532 nm light source with an irradiance of $100\;\mathrm{mW}/cm^{2}$ was employed in this simulation. Light propagation within blood vessels and tissue is modeled using the diffusion approximation,^26^ which is well established for modeling light transport in scattering-dominated media such as biological tissue:

$$\frac{\partial \psi }{\partial t}-D_{\text{opt}}\nabla^{2}\psi +c_{*}\mu_{a}\psi =0,$$ (1)

where ψ is the photon fluence rate ($\mathrm{mW}/cm^{2}$), $c_{*}$ is the speed of light in tissue, $\mu_{a}$ is the absorption coefficient of the medium (epidermis, dermis, or blood), and $D_{\mathrm{opt}}$ is the optical diffusion coefficient defined as

$$D_{\mathrm{opt}}=c_{*}/[3(\mu_{a}+(1-g)\mu_{s})],$$ (2)

where $\mu_{s}$ is the scattering coefficient, and g is the anisotropy factor of the tissue. The photon source is modeled as an incident photon flux on the tissue surface, described by the following boundary condition:

$$(1-r)P(t)c=-D_{\text{opt}}\overset{\to }{n}\cdot \frac{\partial \psi }{\partial y},$$ (3)

where r is the internal reflection coefficient,^26^ which was calculated to be 0.034 based on the refractive-index mismatch between air and skin,^27^ P(t) is the incident irradiance, c is the speed of light in a vacuum, and $\vec{n}$ is the photon flux gradient in the normal direction.

The optical parameters used in the simulation are listed in Table 1.^27^^–^^29^

**Table 1:** Parameters for light transport.

	Optical parameters^27^			
	$\mu_{a}$ (${\mathrm{cm}}^{-1}$)	$\mu_{s}$ (${\mathrm{cm}}^{-1}$)	g	n
Stratum Corneum	181	2200	0.925	1.45
Epidermis	1.1	184.4	0.774	1.4
Dermis	2.6	184.4	0.774	1.4
Blood	206.2	500	0.98	1.33

### 2.3 Oxygen Diffusion and Reaction

Oxygen delivery to the tissue occurs via two primary pathways: diffusion from the vasculature and transcutaneous diffusion, and this process can be described by the following equation defining the tissue diffusion coefficient.

$$\frac{\partial C_{o}}{\partial t}+\nabla \cdot (-D_{o}\nabla C_{o})+U\cdot \nabla C_{o}=\Gamma_{1}+\Gamma_{2},$$ (4)

where $C_{o}$ is the oxygen concentration, $D_{o}$ is the diffusion coefficient of oxygen, U is the velocity flow vector of the blood, $\Gamma_{1}$ is the metabolism consumption of oxygen at 1.7 μmol/(L·s),^28^ and $\Gamma_{2}$ is the rate of oxygen consumption in PDT. For modeling purposes, a linear relationship between molecular oxygen consumption and ROS generation is assumed. This simplification implies that all oxygen molecules consumed are theoretically converted into ROS. Therefore, the oxygen consumption rate is taken to be equal to the ROS generation rate.

$$\prod_{\mathrm{ROS}}=\Gamma_{2}=2.303\varepsilon \Phi C_{m}(t)\psi \frac{C_{o}(t)}{C_{o}(t)+\frac{k_{p}}{k_{ot}}},$$ (5)

where ε is the molar extinction coefficient,^30^ Φ is the quantum yield, Φ=0.6 for HMME, $\frac{k_{p}}{k_{ot}}$ represents the ratio of the photosensitizer triplet-state decay rate to the rate constant for triple-state photosensitizer reacting with triplet ground-state oxygen, reflecting the efficiency of singlet oxygen generation.

The photochemical reaction parameters used in the simulation are listed in Table 2.

**Table 2:** Parameters for photochemical reaction.

	Definition	Value	Reference
$D_{m}$ ($\mu m^{2}/s$)	Diffusion coefficient of HMME	50	28
$D_{o}$ ($\mu m^{2}/s$)	Diffusion coefficient of oxygen	1500	29
$k_{p}/k_{ot}$ (mol/L)	Ratio between $k_{p}$ and $k_{\mathrm{ot}}$	2.5	29
ϕ	Quantum yield of HMME	0.6	30
ε (L/(mol·cm))	Molar extinction coefficient at 532 nm	9252	27
$K_{m}$ (mol/W)	Comprehensive rate constant of photobleaching	66.21	28
$C_{o}$	Oxygen concentration	—	—
$C_{m}$	HMME concentration	—	—
$\Gamma_{1}(\mu \mathrm{mol}/(L\cdot s))$	Metabolism consumption of oxygen	1.7	28
$\Gamma_{2}(\mu \mathrm{mol}/(L\cdot s))$	Oxygen consumption rate during PDT	—	—
$\Gamma_{m}(\mu \mathrm{mol}/(L\cdot s))$	Photobleaching-induced consumption term of HMME	—	—

Our model incorporated two independent oxygen sources: a fixed vascular inlet (100 mmHg) and a controllable transcutaneous source (159, 79.5, or 16 mmHg) at the skin surface. To systematically evaluate the influence of transcutaneous oxygen gradients on PDT outcomes, we engineered normoxia, hypoxia, and anoxia oxygen tension regimens at the epidermal interface to encompass therapeutic efficacy and adverse effects. These values, grounded in gas–liquid equilibrium principles (Henry’s Law), correspond to experimentally achievable environments rather than intrinsic tissue oxygenation levels:^31^

Normoxia group: surface ${\mathrm{pO}}_{2}=159\;\mathrm{mmHg}$. Skin surface oxygen partial pressure (${\mathrm{pO}}_{2}$) was set at 159 mmHg, representing normal oxygenation under atmospheric air exposure ($21\%O_{2}\times 760\;\mathrm{mmHg}$).

Hypoxia group: surface ${\mathrm{pO}}_{2}=79.5\;\mathrm{mmHg}$. Skin surface ${\mathrm{pO}}_{2}$ was reduced to half of the normoxic level, simulating conditions of insufficient local oxygen supply. Such reductions are experimentally achievable when the skin surface is covered with a thin aqueous layer,^32^ which lowers interfacial ${\mathrm{pO}}_{2}$ according to Henry’s law due to the lower oxygen solubility in water.

Anoxia group: surface ${\mathrm{pO}}_{2}=16\;\mathrm{mmHg}$. Represents a severely oxygen-restricted environment, comparable to an atmosphere containing ∼2% $O_{2}$ (similar to hypoxia chambers or anaerobic culture incubators^33^). Under such conditions, the inward diffusion gradient from ambient air is effectively abolished.

The vascular inlet boundary condition for ${\mathrm{pO}}_{2}$ was prescribed at 100 mmHg, corresponding to physiological arterial ${\mathrm{pO}}_{2}$ values in precapillary arterioles. This boundary-driven oxygen flux, governed by Fickian diffusion and hemoglobin-oxygen dissociation dynamics, ensures a progressive decline in capillary.^34^

### 2.4 Pharmacokinetics of HMME

**Fig. 2:**
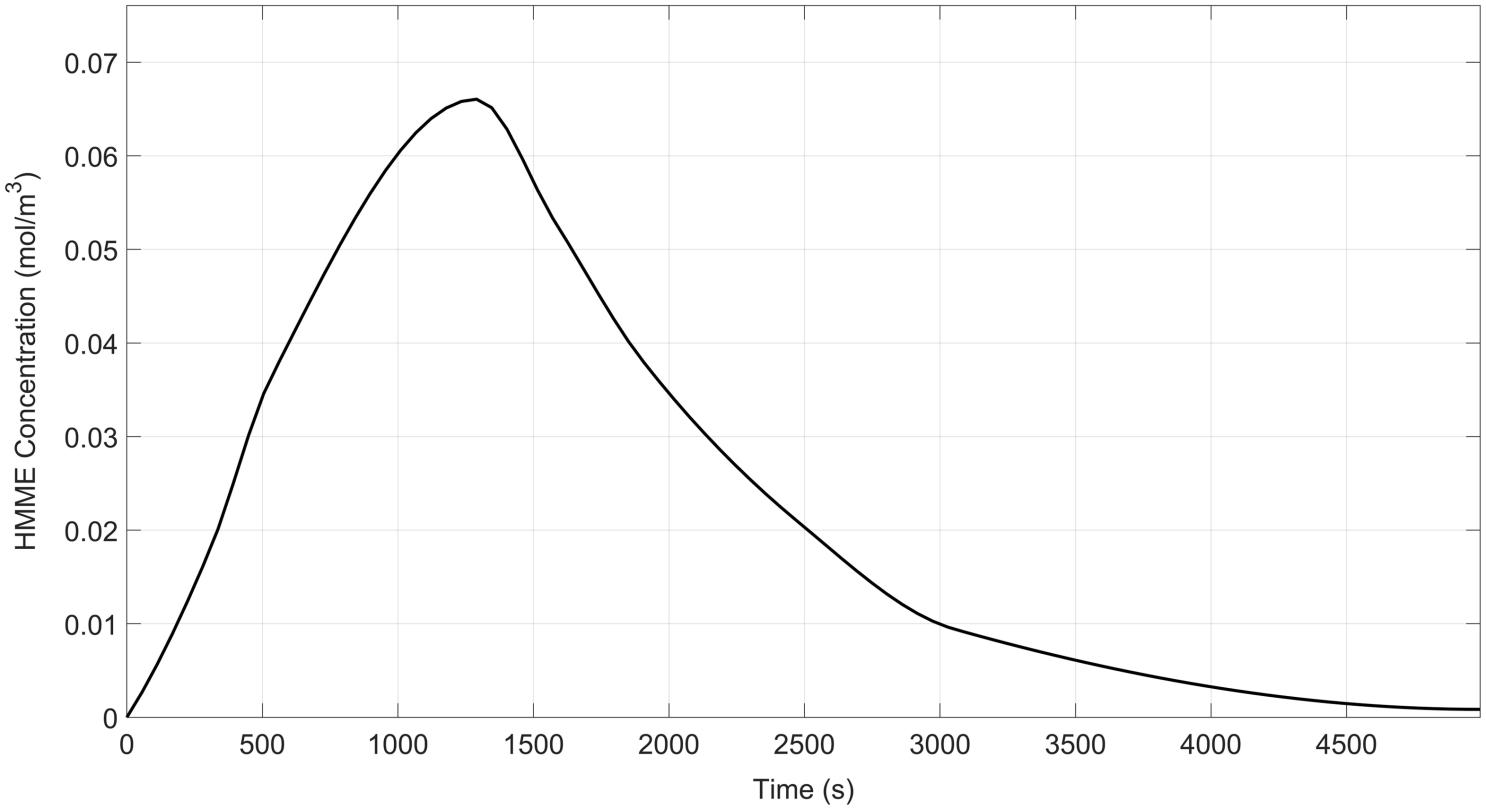
Pharmacokinetics of HMME in blood.

As mentioned above, the photosensitizer of HMME was administered intravenously at a dosage of 5  mg/kg. It was infused for over 20 min using a peristaltic pump.^35^ This “slow bolus” method promotes stable drug circulation, reducing adverse effects from rapid injection, with the corresponding pharmacokinetics shown in Fig. 2.^35^ The HMME was then transported through the circulatory system to the arterioles and eventually delivered to the capillaries and surrounding tissues.

In this simulation, the photosensitizer diffusion and photobleaching in the blood vessels and tissues can be described as

$$\frac{\partial C_{m}}{\partial t}+\nabla \cdot (-D_{m}\nabla C_{m})+U\cdot \nabla C_{m}=\Gamma_{m},$$ (6)

$$\Gamma_{m}=-K_{m}\cdot \frac{C_{o}(t)}{C_{o}(t)+\frac{k_{p}}{k_{ot}}}\cdot \psi \cdot C_{m}^{2}(t),$$ (7)

where $D_{m}$ is the diffusion coefficient of HMME, $C_{m}$ is the photosensitizer concentration, $\Gamma_{m}$ (<0) denotes the photobleaching-induced consumption term of HMME, and $K_{m}$ is the comprehensive rate constant of photobleaching, with a value of 66.21  mol/W.^28^ The main parameters of photosensitizer diffusion and photochemical reactions are also shown in Table 2.

### 2.5 Evaluation Method

To delineate the role of transcutaneous oxygen gradients in modulating PDT-mediated cytotoxicity, four parameters were defined to evaluate the spatiotemporal concentration, accumulation of ROS, and their potential impact on tissue:

#### 2.5.1 Damage threshold

The spatially averaged time integral of the singlet oxygen concentration within the vascular wall region has a damage threshold of a value of $0.222\;\mathrm{mol}/m^{3}$ in our simulations, based on observations of the effective dose required for causing vascular destruction after a standard 20-min treatment. To characterize the region where therapeutic effects are achieved, a ROS accumulation concentration of 80% of this threshold is set as the damage state to determine the extent of damage. The time at which this damage threshold is reached is defined as the end of the treatment.

#### 2.5.2 Photochemical pain threshold

The spatially averaged rate of ROS generation in the papillary dermis during standardized phototherapy exposure (10-min phase) was quantitatively defined as the photochemical pain threshold, specifically referring to the critical threshold where human subjects exhibited clinically validated onset of nociceptive response.^36^ In our simulation, the accurate value of photochemical pain threshold is $9.413\times 10^{-5}\;\mathrm{mol}/(m^{3}\cdot s)$.

#### 2.5.3 Photochemical pain duration

The photochemical pain duration was operationally quantified as the temporal interval spanning from the initial attainment of photochemical pain threshold to subsequent damage threshold in the vascular wall, demarcating the clinically observable window of sustained pain perception during PDT.

#### 2.5.4 Phototherapeutic selectivity coefficient

The phototherapeutic selectivity coefficient (PSC) was operationally defined as the quotient between the ROS tolerance limit in the dermal vascular plexus and the mean cumulative ROS burden within the epidermal compartment, expressed as:

$$\mathrm{PSC}=\frac{\frac{1}{V_{\text{wall}}}\int_{V_{\text{wall}}}\int_{0}^{t_{\text{threshold}}}\prod_{{\mathrm{ROS}}_{\text{wall}}}(t){\mathrm{dtdV}}_{\text{wall}}}{\frac{1}{V_{\text{papillary}}}\int_{V_{\text{papillary}}}\int_{0}^{t_{\text{threshold}}}\prod_{{\mathrm{ROS}}_{\text{papillary}}}(t){\mathrm{dtdV}}_{\text{papillary}}},$$ (8)

where $t_{\text{threshold}}$ denotes the time at which the ROS concentration within the vascular wall first reaches the vascular damage threshold, i.e., the onset of irreversible photochemical injury in the targeted vessel region.

This index serves as a quantitative biomarker assessing treatment specificity, where elevated index values directly correlate with enhanced target–nontarget discrimination efficacy.

These parameters constitute a spatiotemporal quantification framework for ROS kinetics in PDT, mechanistically linking dermal ROS gradients to the nociceptive-tissue injury continuum.

## 3 Results

### 3.1 Spatial Distribution of Light

**Fig. 3:**
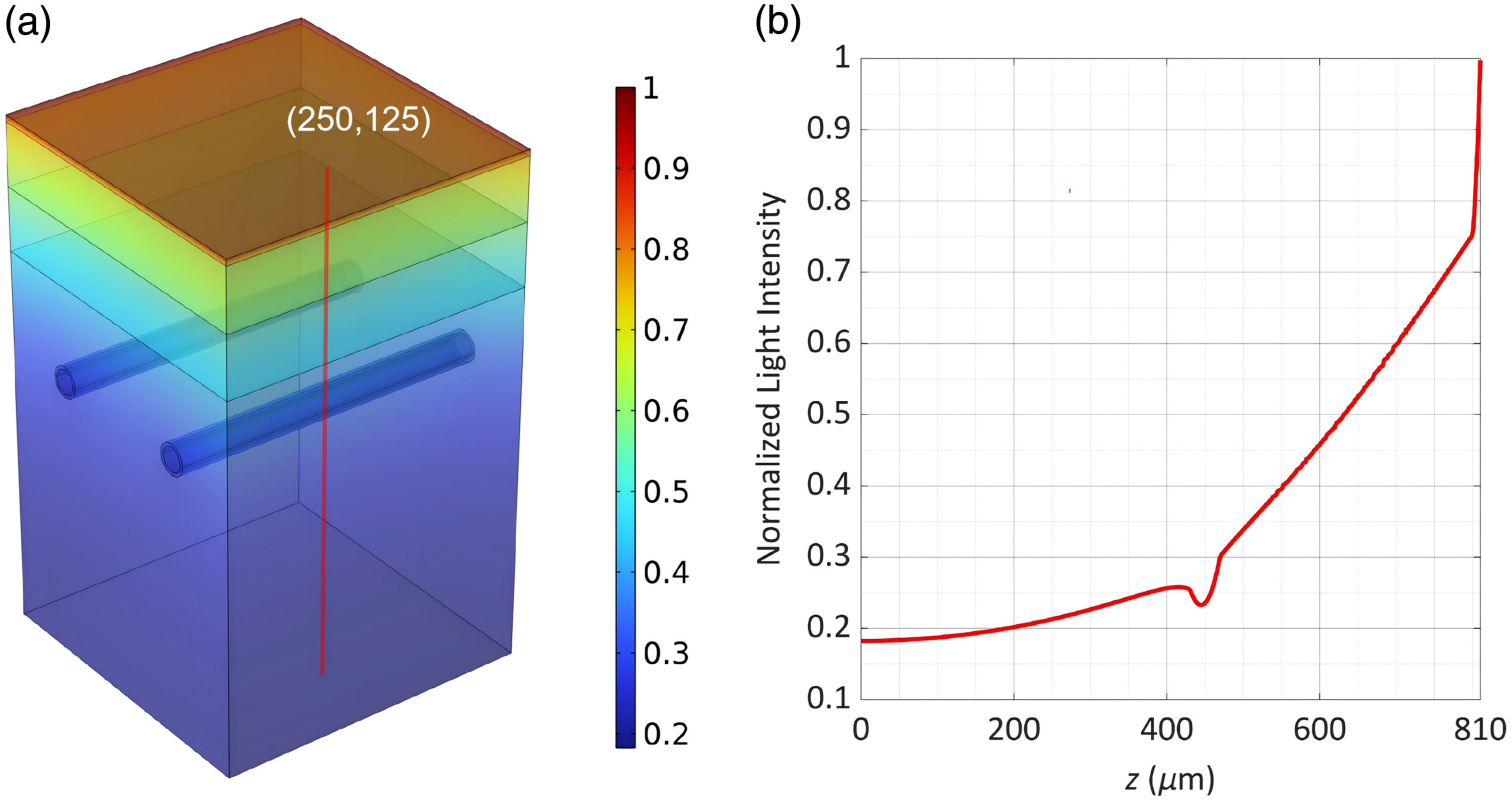
Light distribution in tissue. (a) Normalized 3D light fluence distribution, (b) vertical profile along the red line.

Figure 3 demonstrates depth-dependent photonic flux attenuation kinetics in human skin, characterized by stratum-specific optical property gradients. The stratum corneum exhibits rapid fluence decay due to its elevated absorption coefficient and pronounced scattering anisotropy. Transitioning to the viable epidermis, preserving 59% of incident irradiance at the dermal–epidermal junction. As it subsequently passes through the microvasculature of the papillary dermis, it undergoes significant attenuation due to the strong absorption by hemoglobin, with the light intensity further dropping to 25%. Progressive photon diffusion through reticular dermal collagen matrices culminates in 20% baseline fluence survival at 191  μm height, establishing quantifiable depth-fluence correlations critical for therapeutic window optimization.

Although light energy can penetrate to the vascular layer, the intensity of the light reaching the vascular zone is only 30% of the initial intensity. Therefore, precise generation of ROS in the blood vessels must be achieved by coordinating the spatial and temporal distribution of oxygen concentration and photosensitizer, to enhance the PSC.

### 3.2 Spatiotemporal Distribution of Photosensitizer

**Fig. 4:**
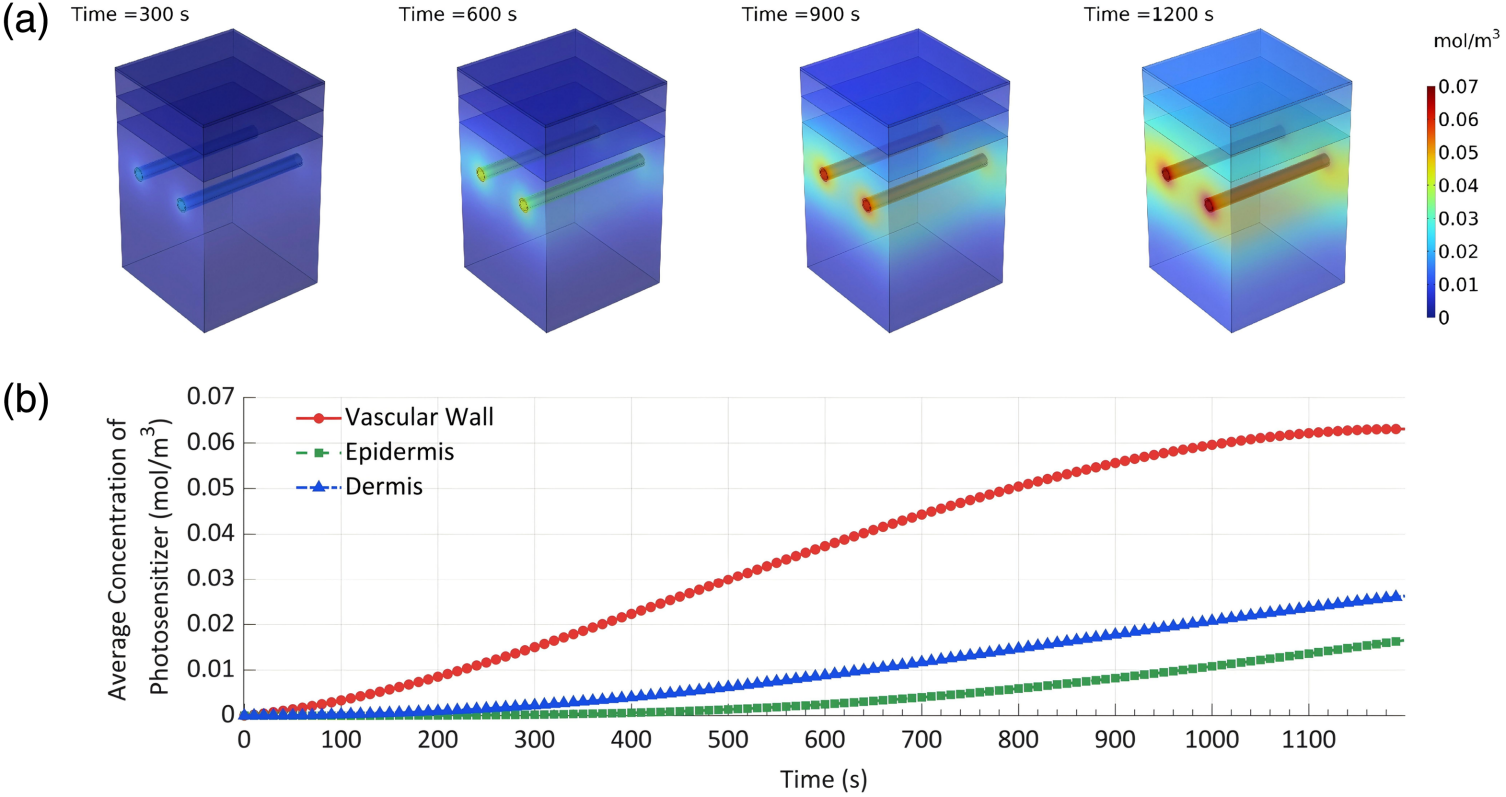
Spatiotemporal distribution of photosensitizer. (a) Three-dimensional distribution at a specific time, (b) mean concentration in different regions.

As shown in Fig. 4(a), the photosensitizer of HMME exhibits significant selective characteristics in its spatiotemporal distribution within tissues, diffusing from the arteriolar inlet into surrounding tissues. Over time, the photosensitizer diffuses from the blood vessels and spreads outward, demonstrating remarkable dynamic evolution characteristics.

At the time of 300 s, the photosensitizer mainly concentrates near the arteriolar inlet with low concentrations, where the peak value remains below $0.018\;\mathrm{mol}/m^{3}$. By the time of 600 s, the photosensitizer concentration at the arteriolar inlet increases to $0.041\;\mathrm{mol}/m^{3}$ and begins diffusing into the surrounding tissues. At the time of 900 s, the photosensitizer concentration near blood vessels continues to rise, reaching $0.061\;\mathrm{mol}/m^{3}$ at the arteriolar inlet, whereas the epidermal concentration also begins to increase to $0.007\;\mathrm{mol}/m^{3}$. By the time of 1200 s, the photosensitizer concentration at arteriolar inlet peaks above $0.067\;\mathrm{mol}/m^{3}$, with the surrounding distribution showing significant concentration gradients, and the concentration in the epidermis noticeably increases to $0.016\;\mathrm{mol}/m^{3}$.

As shown in Fig. 4(b), the photosensitizer selectively accumulates on blood vessel walls, with vessel wall concentrations significantly higher than in the epidermis and dermis. At 1200 s, the photosensitizer concentration in vessel walls is 240% of that in the epidermis and 381% of that in the dermis. This demonstrates that the photosensitizer’s spatiotemporal distribution in three-dimensional space undergoes a clear dynamic evolution process: initially relatively uniform, then progressively accumulating around blood vessels, ultimately forming high-concentration enrichment zones near blood vessels. This phenomenon provides important evidence for further research into the efficiency and mechanisms of photosensitizers during light irradiation or chemical reactions.

### 3.3 Spatiotemporal Distribution of Oxygen

**Fig. 5:**
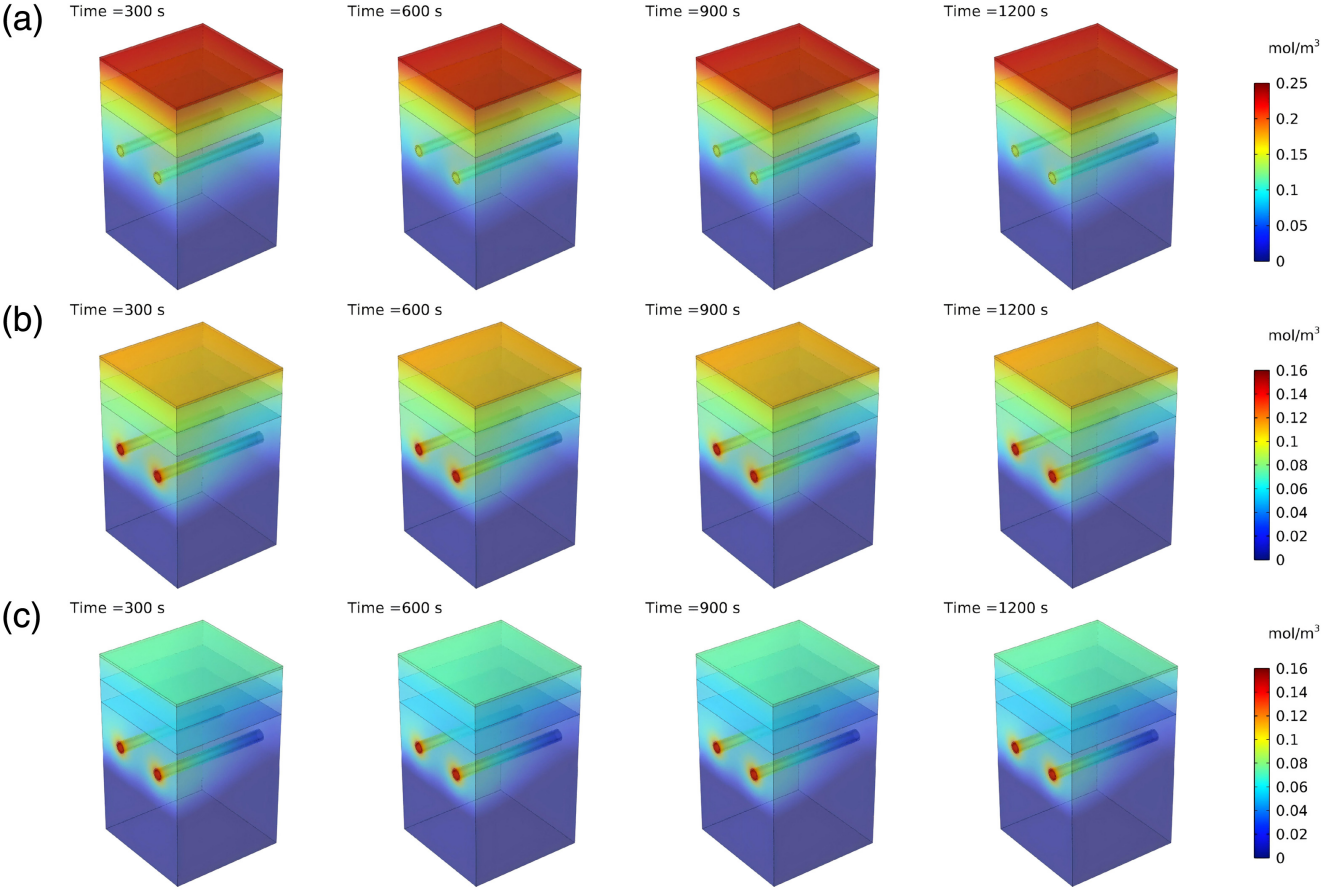
Spatiotemporal distribution of oxygen in tissues under (a) normoxia, (b) hypoxia, and (c) anoxia.

As shown in Fig. 5, significant differences were also observed in the spatial distribution characteristics of oxygen among the groups, whereas the temporal dynamic distribution features showed minimal changes, with limited variation in oxygen spatial distribution over time. The spatial distribution of tissue oxygen concentration demonstrated marked microenvironmental specificity across physiological oxygen gradients, revealing three distinct organizational patterns:

In the normoxia group, the skin surface reached a peak oxygen concentration of $0.232\;\mathrm{mol}/m^{3}$, whereas the arteriolar inlet maintained $0.146\;\mathrm{mol}/m^{3}$, establishing a bidirectional oxygen gradient from epidermis-to-deep tissue and vasculature-to-periphery.

In the hypoxia group, cutaneous oxygen dropped to $0.116\;\mathrm{mol}/m^{3}$ versus $0.146\;\mathrm{mol}/m^{3}$ at arteriolar inlets. The attenuated dermal oxygen diffusion became functionally equivalent to microvascular oxygen delivery, resulting in a vasculature-dominant distribution pattern.

In the anoxia group, although the oxygen concentration further declined, arteriolar inlets preserved $0.146\;\mathrm{mol}/m^{3}$ concentrations. The system completely transitioned to a vasculature-centric distribution, with oxygen selectively accumulating around arteriolar before diffusing to venular outlets and adjacent tissues.

### 3.4 Spatiotemporal Distribution of ROS

**Fig. 6:**
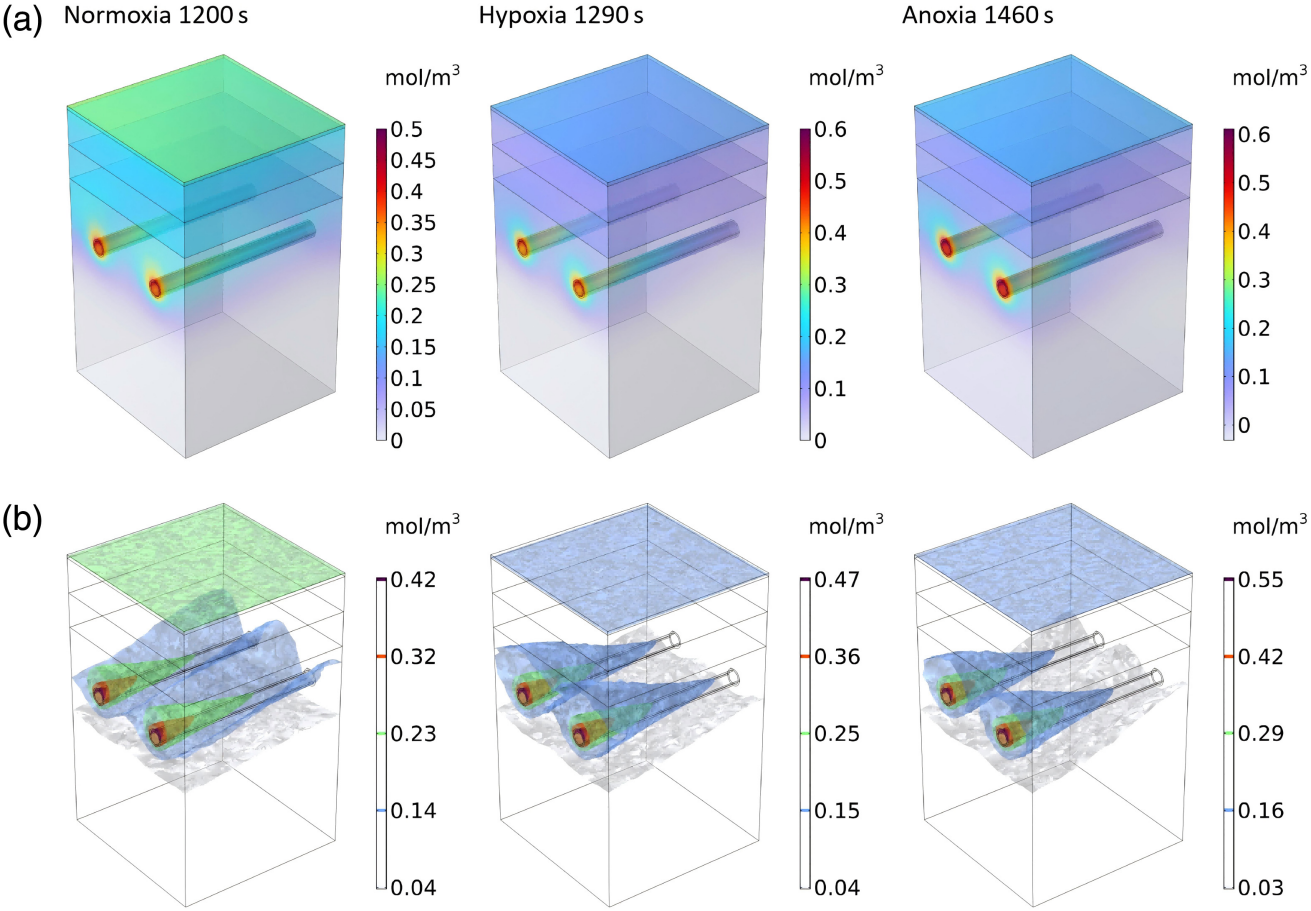
Spatiotemporal distribution of cumulative ROS concentration for normoxia at 1200 s, hypoxia at 1290 s, and anoxia at 1460 s under render mode of (a) surface and (b) isosurfaces.

Figure 6 illustrates cumulative ROS concentration spatial distribution patterns under varying surface oxygen concentrations. All experimental groups exhibited preferential ROS accumulation in peri-vascular regions, particularly concentrated near targeted vasculature.

In the normoxia group, cumulative ROS concentration was predominantly localized in both the targeted vasculature and the epidermal surface. Peak concentrations ($0.463\;\mathrm{mol}/m^{3}$) occurred at the arteriolar inlet, with stratum corneum accumulation exceeding $0.184\;\mathrm{mol}/m^{3}$. The vascular-to-epidermal interface maintained moderate ROS levels (0.146 to $0.285\;\mathrm{mol}/m^{3}$), displaying minimal concentration gradients. Despite maximal ROS reaction, the spatial distribution demonstrated limited targeting specificity.

In the hypoxia group, ROS accumulation became vasocentric, maintaining equivalent arteriolar inlet peaks ($0.524\;\mathrm{mol}/m^{3}$). Epidermal ROS decreased significantly ($>0.092\;\mathrm{mol}/m^{3}$ at stratum corneum) compared with normoxic conditions, forming steep peri-vascular gradients. The temporal threshold for vascular wall damage (defined as $0.222\;\mathrm{mol}/m^{3}$ mean concentration) increased from 1200 s (normoxia) to 1290 s, indicating delayed nontarget diffusion. Correspondingly, PSC increased from 1.31 under normoxic conditions to 1.93 under hypoxic conditions, confirming a substantial enhancement in treatment spatial selectivity.

In the anoxia group, ROS distribution became strictly unipolar, confined to targeted vessels with preserved arteriolar inlet maxima ($0.6\;\mathrm{mol}/m^{3}$). Peri-vascular gradients intensified, whereas epidermal ROS decreased below detectable thresholds. The vascular damage threshold arrival time extended further to 1460 s, demonstrating near-complete spatial confinement of ROS to microcirculatory structures. Correspondingly, PSC increased to 2.10 under anoxic conditions, compared with 1.93 in hypoxia, confirming a further enhancement in spatial selectivity with increasing oxygen restriction.

Overall, the oxygen gradient critically modulates ROS spatial dynamics: Under normoxic conditions, maximal ROS reaction occurs but shows compromised spatial specificity owing to extensive diffusional spread. When cutaneous oxygen availability becomes restricted under hypoxic conditions, ROS redistribution exhibits vascular tropism, with pronounced perimicroarterial selectivity. Remarkably, anoxia microenvironments preserve this vascular targeting paradigm while achieving amplified accumulation fidelity, effectively minimizing off-target deposition through oxygen-dependent confinement mechanisms.

### 3.5 Effect of Oxygen Concentration on the Therapeutic Zone

**Fig. 7:**
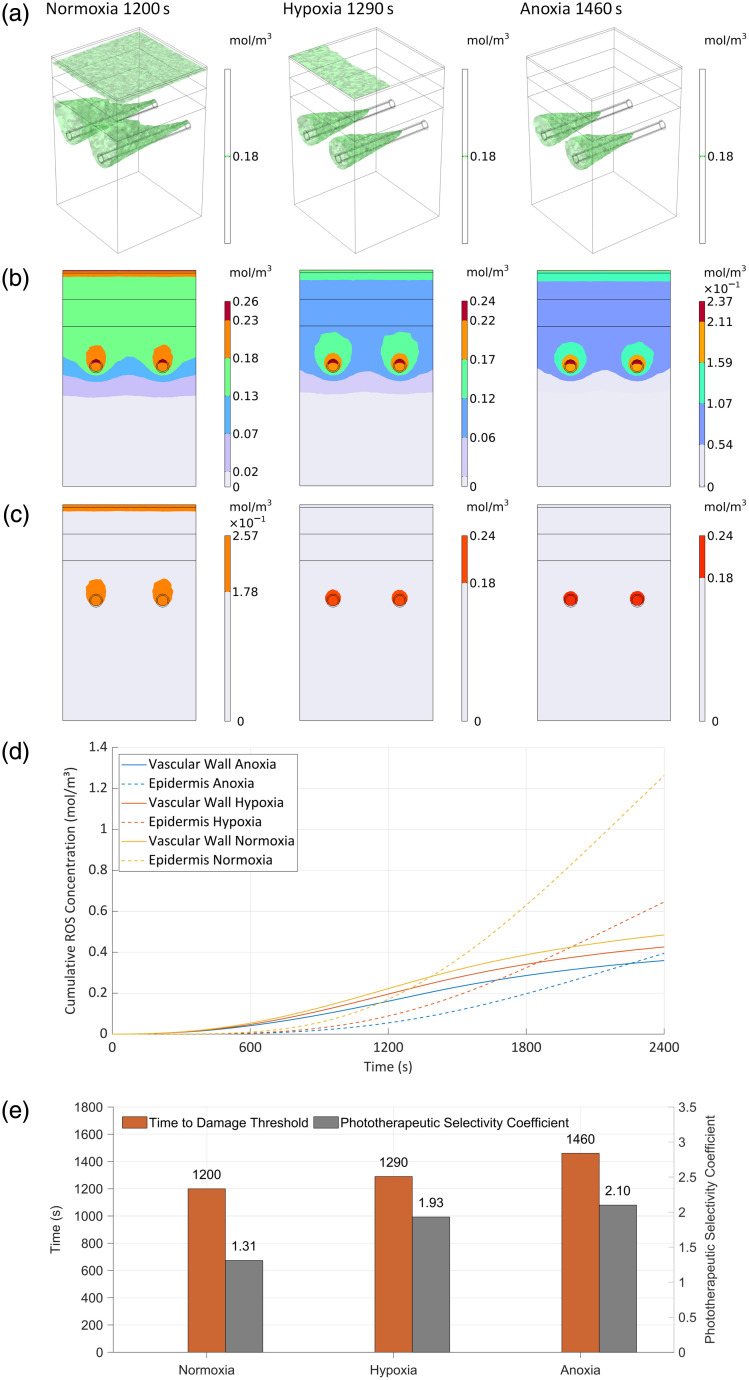
Therapeutic zone spatiotemporal evolution of ROS concentration under three surface oxygen conditions. (a) Therapeutic zone in 3D, (b) cross-sectional ROS distribution, (c) therapeutic zone in cross section, (d) temporal evolution of the spatially-averaged cumulative ROS concentration, (e) damage evaluation parameters values.

Figures 7(a)–7(c) present cross-sectional analyses (y-z plane at x=250  μm) comparing PDT efficacy across oxygen microenvironments, revealing significant spatiotemporal heterogeneity in ROS distribution patterns. The therapeutic zone is specifically defined as the tissue region where the cumulative ROS concentration has reached or exceeded the damage threshold required for a PDT effect.

In the normoxia group, ROS exhibited bimodal accumulation, with primary foci at arteriolar luminal surfaces (peak: $0.463\;\mathrm{mol}/m^{3}$) and secondary epidermal deposition ($>0.146\;\mathrm{mol}/m^{3}$). A radially decreasing gradient extended from vasculature to stratum corneum, correlating with diffuse lesion areas encompassing full vessel diameters and partial epidermal regions. This indiscriminate distribution suggests oxygen-enhanced ROS mobility, causing collateral tissue damage.

In the hypoxia group, ROS redistribution demonstrated vasocentric optimization, peaking at $0.243\;\mathrm{mol}/m^{3}$ on vascular endothelia with constrained periarteriolar spread. Epidermal concentrations decreased 38% versus normoxia (0.092 to $0.132\;\mathrm{mol}/m^{3}$), concomitant with lesion localization to anterior vessel segments (255  μm from arteriolar inlet). The attenuated epidermal damage profile indicates oxygen-modulated ROS confinement mechanisms.

In the anoxia group, spatial selectivity reached maximal refinement with ROS restricted to luminal microdomains ($0.237\;\mathrm{mol}/m^{3}$ peak). Epidermal accumulation decreased 52% versus hypoxia (0.076 to $0.127\;\mathrm{mol}/m^{3}$). Lesions showed precise spatial confinement (<240  μm from arteriolar inlet), achieving complete vascular sparing despite reduced therapeutic scope.

Figure 7(d) illustrates the temporal evolution of average ROS concentrations in vascular and epidermal regions under three conditions. The vascular region exhibited a biphasic pattern in ROS concentration changes, characterized by an initial increase followed by a subsequent decrease. By contrast, the epidermal region demonstrated a gradual, sustained increase in ROS accumulation. The time required for cumulative ROS concentrations to equilibrate between epidermal and vascular walls differed significantly among groups: 1390 s under normoxic conditions, 1860 s under hypoxic conditions, and 2260 s under anoxic conditions. These findings indicate that reduced surface oxygen concentration substantially delays nontargeted ROS accumulation, thereby enhancing PSC.

Figure 7(e) presents comparative therapeutic efficacy parameters across experimental groups. Using consistent thresholds for ROS-induced injury ($0.222\;\mathrm{mol}/m^{3}$) and photochemical pain response ($9.413\times 10^{-5}\;\mathrm{mol}/(m^{3}\cdot s)$), quantitative analysis revealed significant intergroup differences: the normoxia group exhibited a PSC of 1.31, which increased by 48% under hypoxic conditions and by 61% under anoxic conditions. These results demonstrate an inverse relationship between surface oxygen concentration and PSC, with vascular ROS accumulation becoming more pronounced as ambient oxygen levels decreased. The observed negative correlation between PSC and surface oxygen concentration further supports the oxygen-dependent selectivity mechanism.

In summary, oxygen concentration on the skin surface significantly affects the volume and shape of the therapeutic zone. Under hypoxic or anoxic conditions, ROS are more concentrated in the targeted vessels, and the epidermis is less affected, thus significantly increasing the PSC and improving the safety of treatment, although this strategy may come at the cost of reduced injury coverage. The results provide a quantitative basis for optimizing PDT regimens and achieving enhanced PSC.

### 3.6 Effect of Oxygen Gradient on Pain Management

**Fig. 8:**
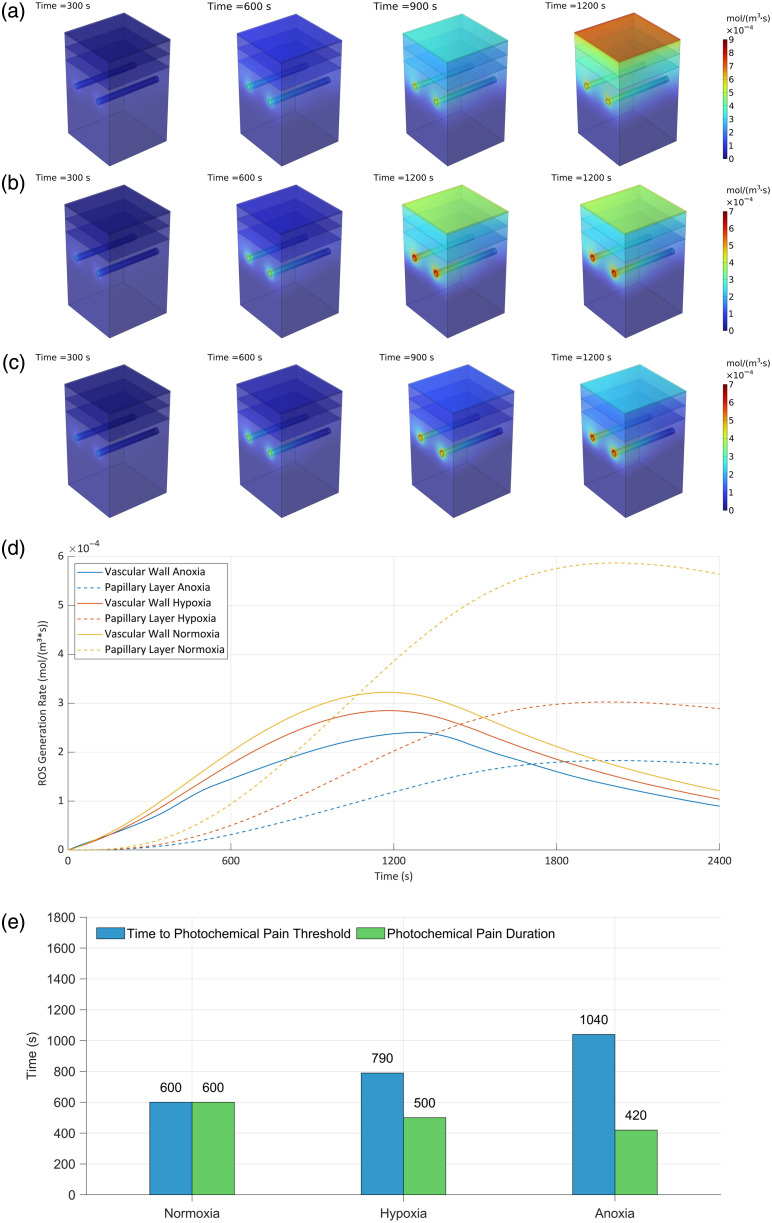
Evolution of ROS generation rate under three surface oxygen conditions. (a) Normoxia, (b) hypoxia, (c) anoxia, (d) average generation rate over time of the typical zone, and (e) pain evaluation parameters values.

Figure 8 illustrates the spatiotemporal evolution of ROS concentration dynamics under three oxygen conditions, revealing distinct stage-dependent and heterogeneous distribution patterns.

During the initial treatment phase (0 to 600 s), intergroup differences in the spatial distribution of ROS generation rates were minimal, with no significant modulation observed. Subsequent progression of treatment revealed a gradual emergence of oxygen concentration regulation on ROS generation rate, demonstrating temporally evolving control mechanisms.

At the 900 s timepoint, all experimental groups reached peak ROS generation rates of $5.613\times 10^{-4}\;\mathrm{mol}/(m^{3}\cdot s)$ at the arteriolar inlet. However, significant intergroup divergence was observed in epidermal ROS generation rates. Quantitative analysis revealed that the normoxic group maintained 64% of the arterial inlet generation rate at the skin surface, whereas hypoxic conditions reduced cutaneous ROS generation rate to 34% of vascular levels. Under anoxia, surface ROS generation rate further diminished to 23%, indicating a pronounced oxygen-dependent gradient with preferential perivascular ROS accumulation.

At 1200 s, a significant spatial redistribution of ROS generation rate patterns was observed. In the normoxia group, the maximal generation rate relocated to the epidermal surface, reaching $8.204\times 10^{-4}\;\mathrm{mol}/(m^{3}\cdot s)$, whereas arteriolar inlet rates stabilized at 82% of surface levels. This configuration established a bidirectional concentration gradient, exhibiting progressive decay both: (i) radially from dermal microvasculature into perivascular tissues, and (ii) vertically from the skin surface toward subcutaneous layers. By contrast, both hypoxic and anoxic conditions sustained peak ROS generation rates at arteriolar inlets ($6.744\times 10^{-4}\;\mathrm{mol}/(m^{3}\cdot s)$). The epidermal ROS generation rate in hypoxia specimens recovered to 64% of vascular peak values, whereas anoxic conditions suppressed the cutaneous generation rate to 40% of vascular levels, confirming oxygen-dependent polarization toward vascular microdomains.

Figure 8(d) presents a systematic analysis of spatiotemporal ROS generation dynamics across vascular and papillary layer microdomains under varying oxygen tensions. Using the normoxic condition’s average ROS flux in the papillary layer ($9.413\times 10^{-5}\mathrm{mol}/(m^{3}\cdot s)$ at 600 s) as the photochemical nociception threshold, we identified oxygen-dependent modulation of redox activity. The vascular ROS generation rate peaked uniformly near 1200 s across all groups, with normoxia specimens exhibiting maximal activity (343% of threshold) at 1170 s, versus hypoxia (303%) at 1180 s and anoxia (256%) at 1290 s. Papillary layer kinetics revealed striking oxygen sensitivity: normoxia peaks reached 624% threshold at 2010 s, whereas hypoxia advanced peak onset to 1990 s with 48% amplitude reduction (322% threshold). Anoxic conditions showed maximal suppression −68% decreased amplitude, 194% threshold) with the latest peak latency (2010 s), confirming oxygen gradient-dependent spatial polarization of redox activity.

These findings indicate that anoxic conditions can consistently maintain ROS levels below 194% of the threshold, suggesting this characteristic may provide new regulatory strategies for reducing pain responses in photodynamic therapy.

Figure 8(e) reveals the changing pattern of pain parameters under different oxygen conditions. In the normoxia group, the first time to reach the photochemical pain threshold was 600 s, and the photochemical pain duration was 600 s. In the hypoxia group, the first time to reach the threshold was delayed to 790 s, and the duration was shortened to 500 s. In the anoxia group, the first time to reach the threshold was 1040 s, and the photochemical pain duration was only 420 s. With the reduction of the surface oxygen concentration, the time to the threshold of the pain showed a linear delay, and the duration of the pain was correspondingly shortened, and the specific relationship was shown in Fig. 8(e).

In summary, lowering the partial pressure of skin surface oxygen not only suppressed the generation rates of ROS in the nontargeted region and reduced the nontargeted damage to tissues but also significantly alleviated the immediate pain caused by photochemical reaction during PDT treatment, shortened the photochemical pain duration, and enhanced the treatment tolerance and overall comfort of patients. This further validates the clinical potential of the oxygen modulation strategy in enhancing the PSC and reducing the risk of side effects.

### 3.7 Effect of Light and Oxygen Synergism

**Fig. 9:**
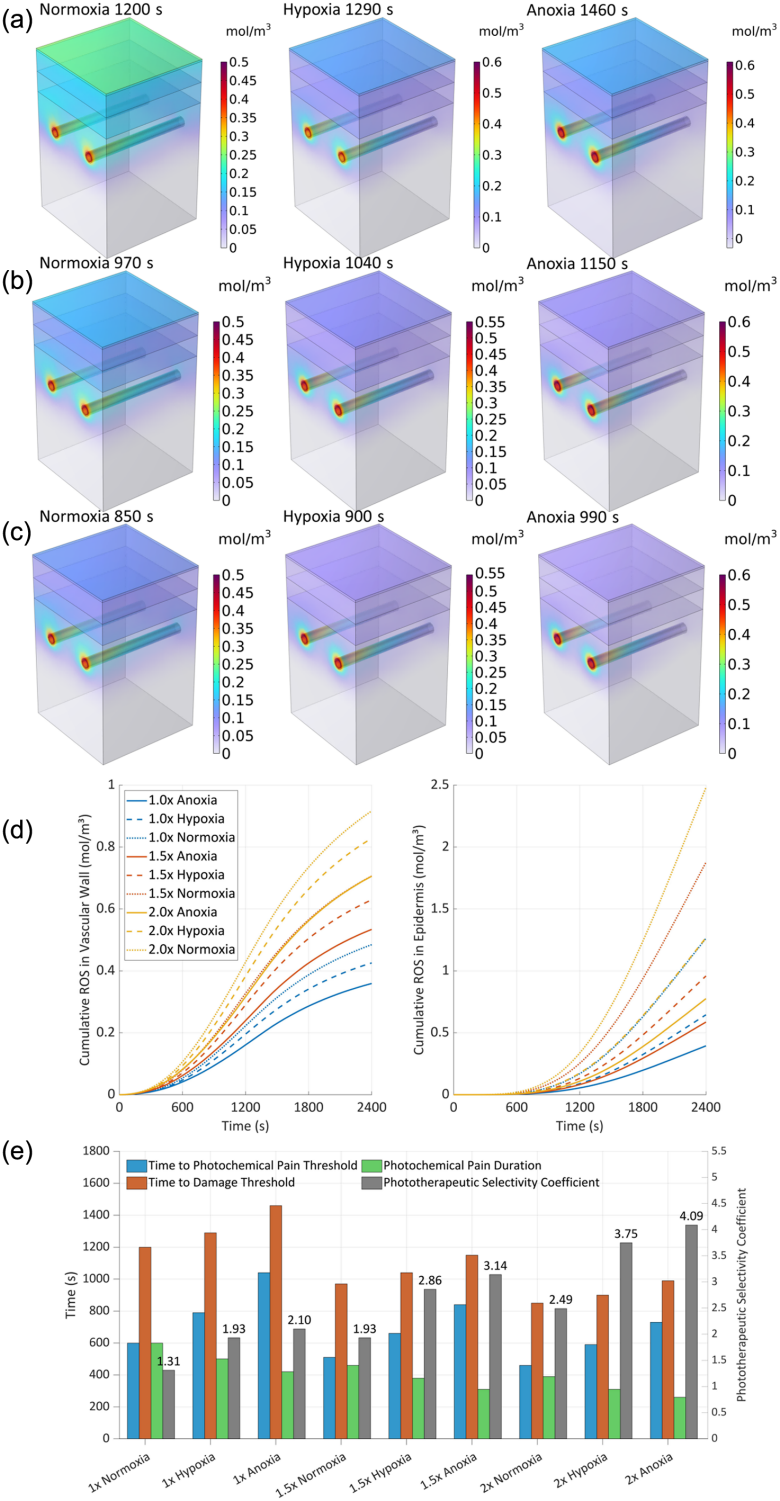
ROS concentration dynamics relationship with light and oxygen synergism. (a) $100\;\mathrm{mW}/{\mathrm{cm}}^{2}$ (1×), (b) $150\;\mathrm{mW}/{\mathrm{cm}}^{2}$ (1.5×), (c) $200\;\mathrm{mW}/{\mathrm{cm}}^{2}$ (2×), (d) average concentration over time of typical zone, and (e) evaluation parameters values.

Figures 9(a)–9(c) exhibit the changing characteristics of ROS distribution under the synergism combination of oxygen concentration of normoxia, hypoxia, and anoxia, and light irradiance of 100, 150, and $200\;\mathrm{mW}/{\mathrm{cm}}^{2}$. The results show that with the increase of light intensity, the accumulation of ROS in the targeted vessel region is more concentrated, the spatial distribution range is narrowed, and a stronger selective distribution in the vessel wall can be achieved. This concentrated distribution led to further enhancement of the PSC.

Figure 9(d) further analyzes the evolution pattern of ROS concentration over time under different light and oxygen conditions. The results showed that the trend of temporal distribution within each light irradiance group was basically consistent with the regulation pattern under the normoxia group. Overall, at the same time point, the higher the light irradiance, the greater the ROS concentration, indicating a positive correlation between the light intensity and ROS generation rate.

Figure 9(e) shows the effects of different lighting conditions and transcutaneous oxygen supply rates on pain kinetics and tissue damage. The data show that light irradiance and oxygen environment present a significant interaction effect on the evaluation indexes:

Light irradiance effect: Compared with the original light irradiance of $100\;\mathrm{mW}/{\mathrm{cm}}^{2}$, increasing light irradiance to 1.5 and 2 times under standard oxygen conditions reduced photochemical pain duration during PDT treatment by 23% and 35%, respectively, whereas the PSC increased significantly by 48% and 90%. The stronger the light irradiance, the more concentrated the ROS generation, and the smaller the therapeutic zone.

Oxygen conditioning effect: Reducing surface oxygen concentration at a fixed light irradiance also modulated pain and injury. For example, at the irradiance of $150\;\mathrm{mW}/{\mathrm{cm}}^{2}$, lowering oxygen concentration shortened photochemical pain duration by 23% and increased PSC by 62%; at the irradiance of $200\;\mathrm{mW}/{\mathrm{cm}}^{2}$, surface anoxic conditions shortened photochemical pain duration by 33% and increased PSC by 65%. It can be seen that lower oxygen concentration can effectively reduce epidermal ROS accumulation and control damage to nontarget tissues, thus optimizing the overall treatment experience.

In summary, choosing the right combination of the light intensity and transcutaneous oxygen supply rate plays an important role in PDT optimization. Increasing light irradiance significantly enhances the generation and aggregation of ROS at the lesion and improves the therapeutic efficiency, whereas decreasing epidermal oxygen supply rate helps to control ROS generation in the epidermal region, reducing pain and shortening the photochemical pain duration. Under the combination of high light intensity and an anoxic environment, ROSs are more concentrated in the vascular region, the concentration in the epidermal region is significantly reduced, the photochemical pain duration may be reduced by 57%, and the PSC is significantly improved by 213%. This fully demonstrates the clinical potential of choosing the right combination of light source irradiance and surface oxygen supply rate in improving the efficacy and patient comfort of PDT.

### 3.8 Effects of Vessel Diameter on PDT Treatment

The modeled capillaries featured a 40  μm inner diameter, 5  μm wall thickness, and 250  μm intervascular spacing between centers. Under physiological conditions, human capillaries with typically ∼10  μm diameter generally extend less than 500  μm in length, with most tissue cells residing within 200  μm of the nearest capillary.^37^ Significant anatomical variations exist in skin tissue structure and vascular distribution across individuals and body regions. This microenvironmental diversity may influence the generalizability of our simulation results, particularly given the diameter-dependent nature of blood flow velocity.

Vessel diameter and blood flow velocity exhibit codependent relationships governed by hemodynamic principles. Flow rate through cylindrical vessels shows:

$$Q=\frac{\Delta P\cdot \pi R^{4}}{8\eta L},$$ (9)

where Q is the flow, ΔP is the differential pressure; R is the vessel radius, η is the blood viscosity, and L. is the vessel length. In the case of laminar flow, the blood flow rate in the vessel is proportional to the fourth power of the vessel radius.

**Fig. 10:**
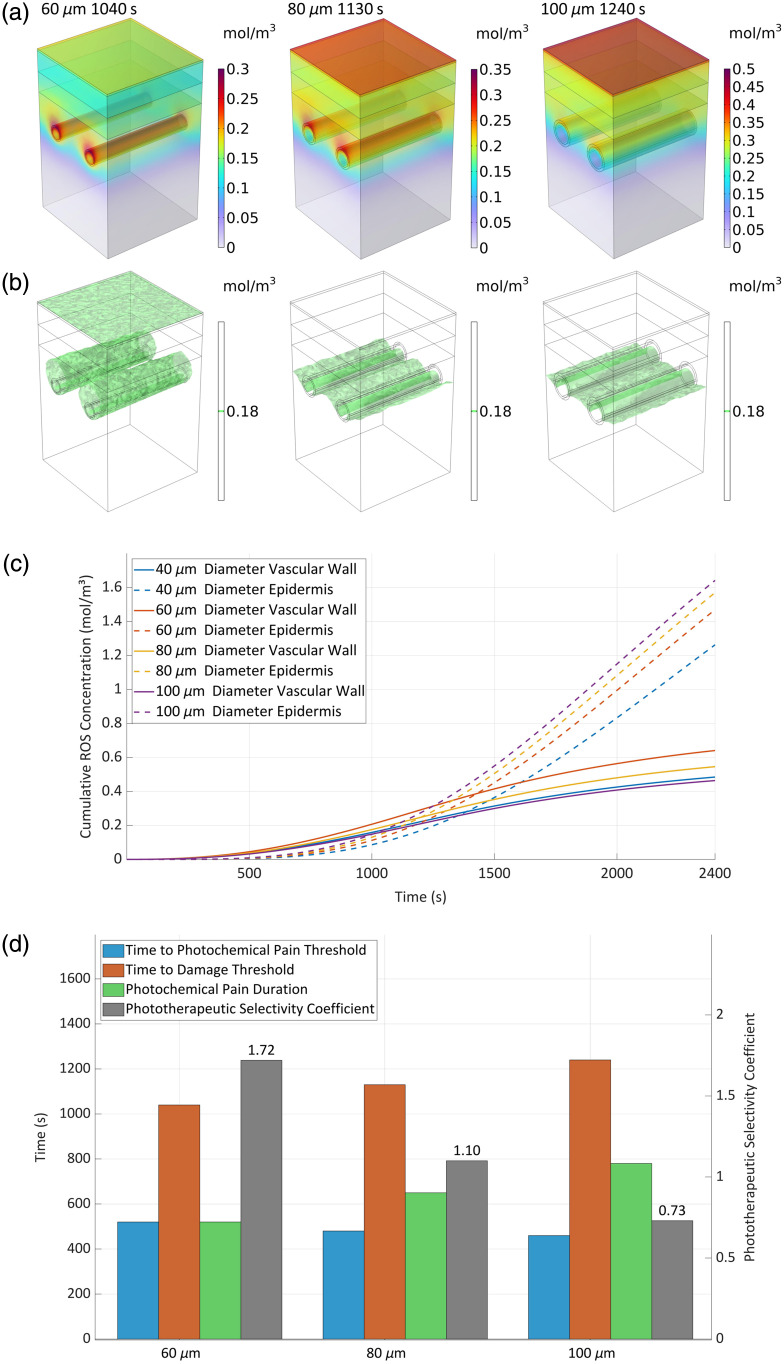
ROS concentration dynamics relationship with vessel diameters. (a) Spatiotemporal distribution, (b) therapeutic zone over threshold, (c) average concentration over time of typical zone, and (d) evaluation parameters values.

Figures 10(a) and 10(b) reveal the dynamic relationship between vessel diameter and the spatial distribution of reactive oxygen species: (1) As the vessel diameter increases, the blood flow velocity is synchronized to increase, and the elevated flow velocity shortens the residence time of the photosensitizer and the oxygen in the local vasculature, which leads to faster diffusion and tends to be uniformly distributed in the axial direction of the vessel, and this phenomenon results in a more uniform distribution of ROS in the axial direction of the vessel. (2) The enrichment of ROS in the region of the upper wall of the blood vessel is particularly significant, mainly due to the relatively high absorption coefficient of light in the blood; with the increase of the vessel diameter and the elevation of the blood flow rate, the selective aggregation of light at the upper wall of the blood vessel is further enhanced, which promotes the local generation of ROS.

Figure 10(c) exhibits the distribution of ROS concentration with time for different vessel diameters and blood flow rates. It can be seen that at the same time, the vessel diameter increases, the deeper the influence of ROS diffusion at the epidermis, and the average ROS concentration value increases, but the average ROS concentration value at the blood vessel, except at the 60  μm diameter condition, decreases for the vessels with increasing diameters.

Mean ROS concentration is co-modulated by dual vasodilation effects: enhanced oxygen supply from accelerated blood flow versus reduced photosensitizer retention due to shortened transit time, whereas increased vessel diameter simultaneously exacerbates the axial disparity in light fluence between superficial and deep vascular regions. For a vessel with a diameter of 60  μm, elevated oxygen availability dominates over reduced photosensitizer retention, increasing the mean ROS concentration. Conversely, for vessels with diameters of 80 and 100  μm, diminished photosensitizer dwell time progressively outweighs oxygen gains, reducing mean ROS levels below those of 60 and 40  μm diameters, with the 100  μm case exhibiting the lowest concentration due to maximal diffusion constraint.

Figure 10(d) reveals that vascular dilation significantly impacts pain-related parameters and tissue damage levels. When vessel diameter increases from normal to the 100  μm diameter condition—corresponding to progression from pink-type to hypertrophic port-wine stains—photochemical pain duration demonstrates an increase of 30%. Conversely, the tissue PSC decreases with vessel dilation, with the 100  μm diameter condition (representing severe hypertrophic port-wine stains) showing 45% reduction compared with normal pink-type lesions.

These findings indicate that vascular dilation may reduce photodynamic therapy PSC while increasing ROS accumulation in the papillary layer. This phenomenon correlates with characteristic vascular abnormalities in port-wine stains and may exacerbate treatment-induced pain responses.

### 3.9 Effect of Blood Vessel Oxygen Supply on PDT Treatment

The skin’s unique position as the body-environment interface creates a dual oxygen supply system, combining atmospheric diffusion and vascular perfusion to establish steep tissue oxygen gradients. Atmospheric oxygen permeates the skin surface through passive diffusion while microcirculatory networks deliver blood-borne oxygen. This transcutaneous oxygen transport reaches depths of 266 to 375  μm, contributing substantially to epidermal and superficial dermal oxygenation.^32^ Reported physiological ${\mathrm{pO}}_{2}$ levels in human tissues range from 23 to 70 mmHg, with cutaneous oxygen tension typically maintained at ∼40  mmHg due to reduced perfusion and lower metabolic demand.^34^

Although arterial ${\mathrm{pO}}_{2}$ typically ranges 80 to 100 mmHg in human tissues,^34^ significant site-specific and interindividual variations occur in vascular oxygenation. To systematically assess photodynamic therapy (PDT) efficacy under varying oxygen conditions, we established three vascular inlet ${\mathrm{pO}}_{2}$ levels (100 mmHg baseline; 90 and 80 mmHg comparative groups) to enable quantitative evaluation of oxygen-dependent therapeutic responses, including ROS reaction dynamics, spatial injury patterns, and physiological parameter fluctuations.

**Fig. 11:**
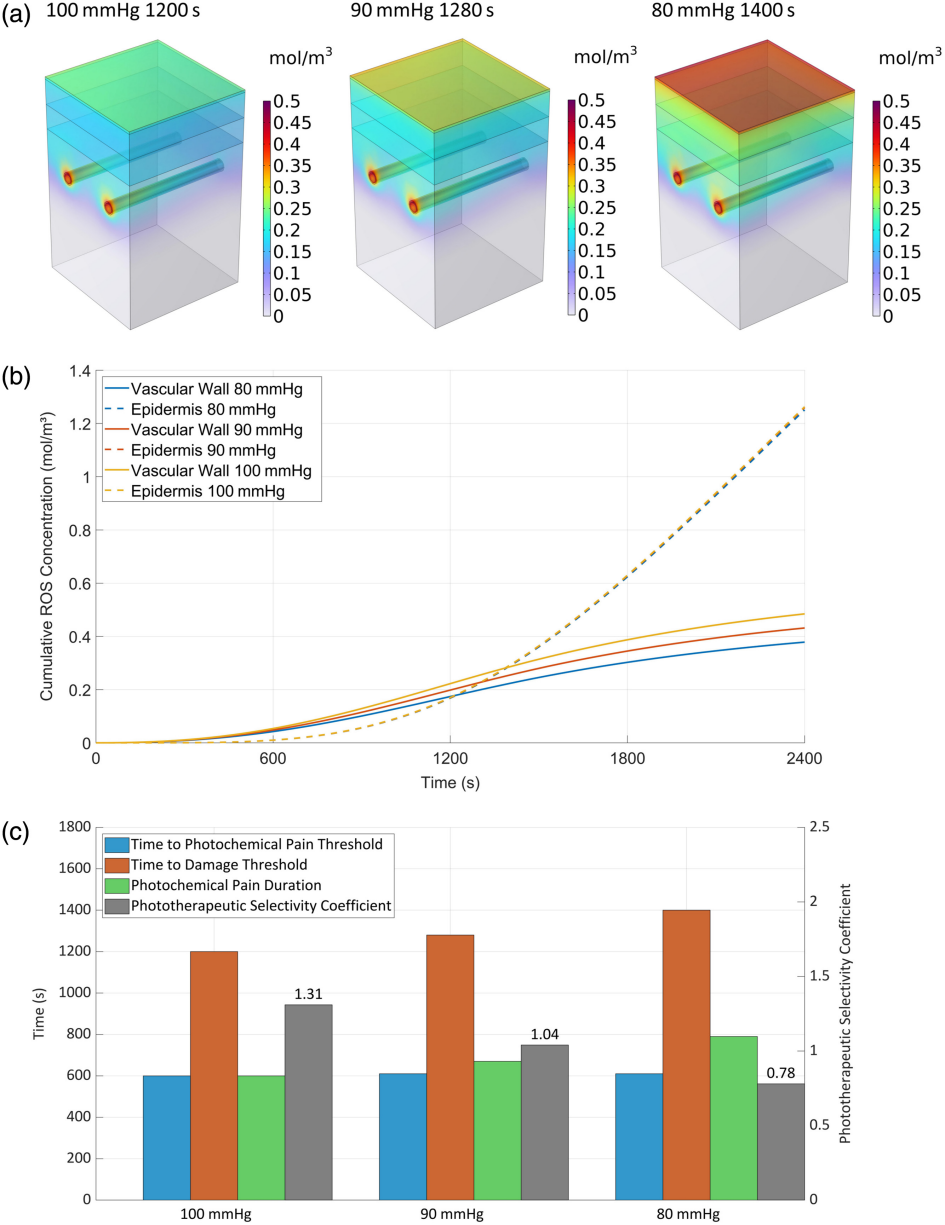
ROS concentration dynamics relationship with blood oxygen level. (a) Spatial distribution, (b) concentration dynamic, and (c) evaluation parameters values.

Figure 11(a) demonstrates the oxygen-dependent spatial regulation of ROS, with perivascular generation rates declining proportionally as arteriolar inlet ${\mathrm{pO}}_{2}$ decreased from 100 to 80 mmHg. Comparative analysis in panel (b) revealed distinct tissue responses: vascular regions exhibited oxygen-dependent ROS concentration (+28% at 100 versus 80 mmHg); epidermal concentrations remained stable (<1% variation), highlighting predominant deep-tissue modulation. Quantitative therapeutic outcomes (c) revealed inverse dose-response relationships ∼21% and 41% prolongation in photochemical pain duration versus 12% and 32% reduction in PSC at 90 and 80 mmHg, respectively.

Arteriolar oxygen partial pressure reduction effectively suppressed deep tissue ROS generation, decreasing overall tissue injury while maintaining epidermal ROS concentrations (<1% variation), thereby increasing the PSC. These findings demonstrate that vascular oxygenation modulation influences treatment penetration depth and photochemical pain duration yet exhibits inferior spatial control compared with epidermal oxygenation strategies in photodynamic therapy. Optimal selectivity for HMME-PDT requires combined oxygenation approaches integrating vascular modulation with superficial control methods.

### 3.10 Effects of Illumination Timing under Different HMME Infusion Protocols

HMME is typically administered via the intravenous route in clinical practice. Reported illumination timing protocols during the injection phase primarily fall into two categories: (1) immediate illumination following intravenous bolus injection;^38^ and (2) constant-rate infusion via a peristaltic pump, with illumination initiated ∼10  min after the cessation of infusion.^9^^,^^35^^,^^39^

This study adopted the second protocol, employing a peristaltic pump for intravenous HMME infusion at a constant rate (∼2 to 5  mL/min) over 20 min. This “slow intravenous bolus” method facilitates the steady entry of the drug into the systemic circulation, thereby mitigating potential local vascular irritation and systemic adverse reactions associated with rapid bolus injection.^40^ The corresponding pharmacokinetic concentration-time profile under this administration mode is shown in Fig. 2.

To systematically evaluate the impact of illumination timing on photodynamic efficacy, we simulated three typical scenarios: (1) illumination coinciding with the start of infusion, corresponding to the phase of rising drug concentration in the blood; (2) illumination 10 min after the infusion onset, corresponding to the period when the intravascular drug concentration approaches its peak; and (3) illumination immediately upon completion of the 20-min infusion, simulating the conventional post-bolus illumination scheme.

**Fig. 12:**
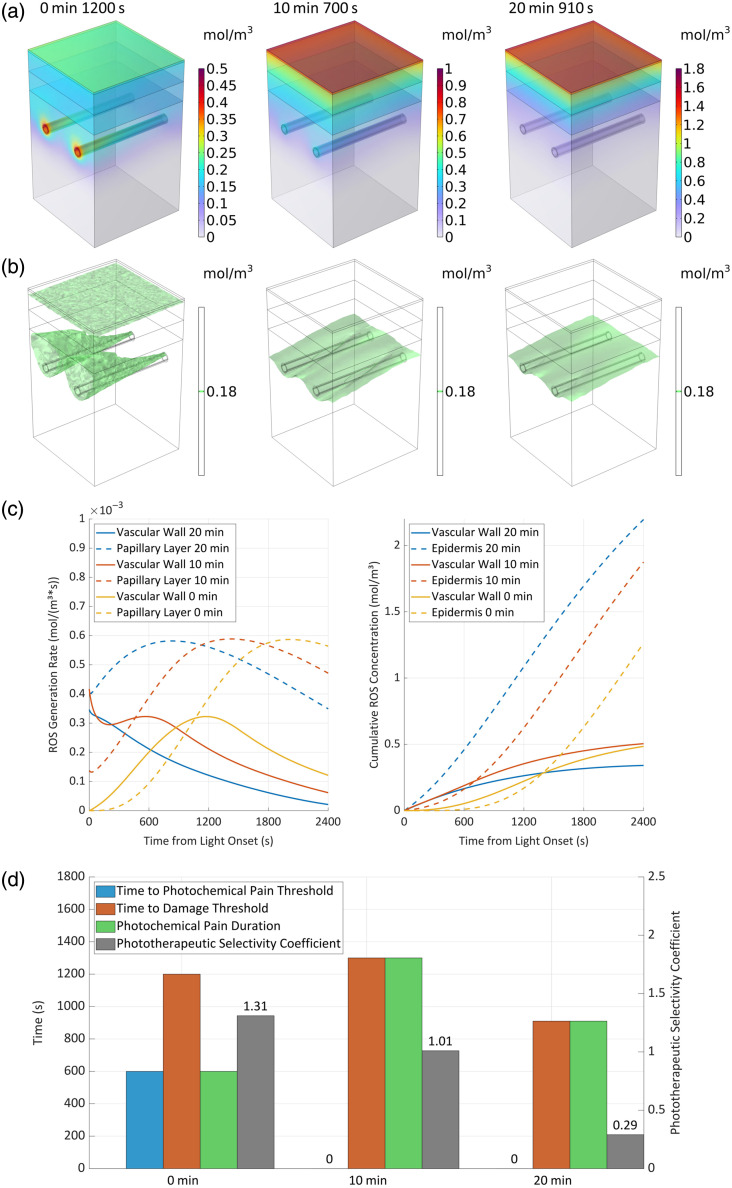
ROS concentration dynamics relationship with illumination timing (a) spatial distribution, (b) therapeutic zone over threshold, (c) concentration dynamics, and (d) evaluation parameters values.

Figures 12(a) and 12(b) elucidate the influence of illumination timing on the spatial distribution of ROS and the resulting tissue damage. Delaying the illumination allowed for extended HMME diffusion within the tissue, leading to higher drug accumulation and consequently, an increase in overall ROS production. Specifically, in the epidermal region, the surface ROS concentration reached ∼0.936 and $1.641\;\mathrm{mol}/m^{3}$ when illumination was delayed by 10 and 20 min, respectively. However, the delayed illumination also reduced the photosensitizer concentration gradient between blood vessels and the surrounding tissue. Although this enabled an expanded photodynamic lesion area for PWS, fully encompassing the epidermis and dermal papillae, it concurrently resulted in a significant loss of PSC.

Figure 12(c) details the ROS generation kinetics in the vessel wall and dermal papillae, along with the corresponding cumulative concentrations in the vessel wall and epidermis. The illumination timing directly determined the initial ROS generation rate. Both 10-min and 20-min delays led to an initial generation rate that exceeded the established pain threshold, which directly contributed to a sharp increase in the cumulative ROS concentration within the epidermis. Quantitatively, compared with immediate illumination (0-min delay), a 10-min delay increased the cumulative concentration in the vessel wall by only 4%, whereas it surged by 49% in the epidermis. It is noteworthy that under the 20-min delay protocol, the average ROS concentration in the vessel wall surpassed that of the immediate illumination group after 1400 s and was ∼30% higher by 2400 s; meanwhile, the concentration in the epidermis remained 75% higher than that in the immediate illumination scheme.

Figure 12(d) provides a comprehensive evaluation of how illumination timing affects PSC and pain perception. The analysis indicates that the protocol corresponding to traditional intravenous bolus injection (20-min delay) exhibited the poorest effect, with its PSC reduced by 79% compared with immediate illumination. The PSC for the 10-min delay protocol was also reduced by 22%. These results confirm that initiating illumination around the peak drug metabolism period enhances the overall ROS concentration and drug utilization efficiency but at the cost of significantly compromised PSC. From a clinical pain management perspective, both delayed illumination protocols are predicted to induce stronger and more prolonged pain sensations compared with the immediate illumination scheme.

## 4 Discussion

Current PDT protocols lack standardized methods for controlled oxygen modulation at the skin surface. However, biomedical research demonstrates established topical oxygen control techniques across related fields,^41^ offering technical benchmarks for potential PDT applications. These include

• Hypoxia simulation in wound healing studies using physical occlusion,^42^ inert gas displacement,^43^ and oxygen-scavenging reactions^42^ to mimic chronic wound microenvironments;
• Localized oxygen regulation in transdermal drug delivery via low-permeability barrier materials;^44^
• Epidermal barrier modeling and 3D tissue culture systems employing controlled oxygen gradients.

These methodologies collectively highlight the viability of targeted oxygen modulation, providing critical insights for optimizing spatial oxygen distribution in PDT.

The vascular shutdown in PWS PDT poses a significant challenge for experimental verification. Recent studies have made significant strides in elucidating the mechanisms of vascular effects in photodynamic therapy. For instance, utilizing advanced near-infrared luminescence imaging and smart molecular beacons, research has managed to directly observe singlet oxygen generation within blood vessels and successfully correlate it with early events such as thrombin activation and vasoconstriction.^45^^,^^46^ However, as a multipathway event involving endothelial damage, vasomotor changes, intravascular coagulation, and perfusion collapse, its complexity hinders direct observation in current animal models such as the mouse dorsal skinfold window-chamber or cockscomb model. This limitation underscores the need for innovative approaches to dissect the underlying mechanisms.

Our future work will focus on experimental validation of the proposed surface oxygen modulation strategy in animal models. Specifically, controlled epidermal oxygen supply and barrier-based oxygen restriction will be implemented during PDT to evaluate their effects on vascular response, ROS distribution, and pain-related indicators. These studies aim to verify the feasibility and efficacy of spatial oxygen regulation under physiologically relevant conditions. Upon successful preclinical validation, this strategy may be further refined via split-face comparison clinical trials toward translational and clinical studies to assess its safety, controllability, and therapeutic benefit in PWS treatment.

## 5 Conclusion

Our computational framework substantiates a dual-target strategy enhancing PSC while mitigating pain for personalized protocols. By introducing the three kinds of oxygen concentration conditions of “normoxia,” “hypoxia,” and “anoxia,” the study found that the moderate reduction of epidermal oxygenation could significantly improve the spatial distribution of ROS in tissues, effectively increase the PSC, and alleviate photochemical pain.

Specifically, the anoxia group had the highest ROS concentration accumulation efficiency in the vascular zone, and the ROS generation rate in the epidermal nontarget zone was significantly decreased, which resulted in an increase in PSC of ∼60% and a decrease in photochemical pain duration of 30% compared with the normoxic condition. The mechanism can be attributed to the fact that with the reduction of epidermal oxygen supply, the oxygen concentration gradient remodeling inhibits superficial ROS generation and promotes enhanced focusing of ROS responses in deep vascular regions. This strategy breaks through the limitation of the traditional PDT regimen, which only relies on the regulation of light dose or photosensitizer concentration, and provides a new dimension of regulation factor for PWS PDT.

In conclusion, choosing the right combination of the light intensity and transcutaneous oxygen supply rate enables spatiotemporal control of photodynamic oxygen activation, potentially optimizing the therapeutic window between PDT efficacy and patient tolerance. Our computational framework substantiates a dual-target strategy enhancing PSC while mitigating pain, and establishing mathematic model for personalized protocols. The translational capacity of this strategy will be assessed in a two-phase investigation: firstly, through rigorous preclinical validation in established animal models, and subsequently, via split-face comparison clinical trials.

## Data Availability

All data in support of the findings of this paper are available within the article.
